# Trauma Exposure and Mental Health Prevalence Among First Aiders

**DOI:** 10.3389/fpsyg.2022.824549

**Published:** 2022-03-07

**Authors:** Charlotte Rowe, Grazia Ceschi, Abdel Halim Boudoukha

**Affiliations:** ^1^Faculté de Psychologie Laboratoire Psychologie des Pays de la Loire, Université de Nantes, Nantes, France; ^2^Department of Psychology, Université de Genève, Geneva, Switzerland

**Keywords:** trauma, mental health, first aiders, PTSD, comorbidity

## Abstract

**Introduction:**

First aiders are commonly exposed to different forms of traumatic event (TE) during their duties, such as Chronic Indirect Vicarious Exposure which refers to an indirect exposure to aversive details of the trauma (APA, 2013). If the psychopathological impact of TE is well documented, the mental health of first aiders remains neglected. Therefore, our main objectives are (i) to study the link between exposure to traumatic events and psychopathological outcomes and (ii) to quantify the rates of mental health disorders among first aiders.

**Method:**

Our sample comprised of 53 volunteer first aiders (21 females and 32 males) with an average age of 32.4 years (*SD* = 13.6 years). Traumatic event exposure and mental health were assessed through a set of validated questionnaires completed online.

**Results:**

Rates of mental health outcomes were higher than within the general population. Females showed higher scores of post-traumatic stress disorder (PTSD) than males. PTSD scores were significantly correlated with all mental issues scores, apart from tobacco use and eating disorders scores. There was a significant correlation between the number of traumatic events and the years of experience. Exposure to traumatic events only correlated with nicotine dependency. No other correlation reaches statistical significance.

**Discussion:**

The scores of all mental health outcomes were high; a surprising result, as volunteer first aiders are thought to be recruited for their strong dispositional cognitive and emotional abilities. The high levels of post-traumatic stress disorder and burnout, along with the prevalent anxiety and depression, highlight the need for greater psychosocial support. Resilience training and peer support would be useful interventions in this group.

## Highlights

- Compared to community samples, first aiders show high levels of mental health issues, although they are considered to be more resilient in the face of traumatic situations than the general population.
- The high prevalence of mental health problems among first aiders highlights the need to develop screening tools and prevention and intervention protocols specifically adapted to their needs.

## Introduction

The literature on mental health after single or multiple traumatic events (TE) strongly suggests that direct or vicarious exposure to such TE can lead to or interfere with many symptom categories. In a range of studies conducted across a variety of communities, these have been described in the context of post-traumatic stress disorder (PTSD; Jeon et al., 2007), of major depressive disorder (MDD; Salcioglu et al., 2007), of general anxiety disorder (GAD; Karatzias et al., 2019), of substance misuse including alcohol and nicotine dependence (Breslau et al., 2003), of eating disorders (Scharff et al., 2021), of obsessive–compulsive disorder (Ceschi et al., 2011) or of dissociative disorders (Schimmenti, 2018).

The community prevalence of MDD is 4.9% (Knoll and MacLennan, 2017) and has been found to be highly comorbid to trauma and PTSD (Kendler et al., 1998). Anxiety often goes hand in hand with depression. The prevalence of GAD in the general population is between 1.9 and 5.1% (Wittchen, 2002) and that of OCD is 3.0% (CilliÇilli et al., 2004). GAD and OCD have been found to be highly correlated with depression (Fierman et al., 1993; Cromer et al., 2007). Ceschi et al. (2011) showed that obsessive–compulsive symptom severity is predicted by exposure to adverse events. Eating disorders have been linked with sexual trauma (Vanderlinden et al., 1993) and other traumatic events (Brewerton, 2007). Lifetime prevalence estimates of The Diagnostic and Statistical Manual of Mental Disorders (fifth ed.; DSM–5; American Psychiatric Association, 2013) of anorexia nervosa, bulimia nervosa and binge eating disorder are up to 9% (Smink et al., 2012).

Alcohol abuse (Green et al., 1985) and nicotine dependence (Anda et al., 1999) have been studied in trauma-exposed populations. The prevalence of alcohol abuse in a community sample is 4.65% (Grant et al., 2004) and that of nicotine dependence 24% (Breslau et al., 2001). Finally, dissociative disorders are strongly related to trauma exposure and PTSD (Briere, 2006). The prevalence of dissociative disorders has been estimated at 9.7% in the general population (Johnson et al., 2006), including depersonalization disorder (0.8%), dissociative amnesia (1.8%), dissociative identity disorder (1.5%) and dissociative disorder not otherwise specified (4.4%).

If the prevalence of mental disorders following a direct or an indirect exposure to trauma seems to be well known in the general populations, less is known among first aiders. This highlights two problems; first, that the PTSD diagnostic criteria changed with the most recent version of the DSM (American Psychiatric Association, 2013), which added two new forms of trauma exposure (learning that a relative or close friend was exposed to trauma); Indirect exposure to adverse details of trauma, usually in the course of professional duties (e.g. first responders and medics). Little research has been devoted to the impact of those last two forms of trauma exposure and less is known about their impact in terms of the prevalence of mental disorders. Research has shown that the more frequent the exposure to traumatised individuals, the higher the risk of vicarious trauma (Saakvitne and Pearlman, 1996). Understanding psychopathological processes allow us to better prevent, diagnose and treat trauma-related mental ill health (Al Falasi et al., 2021).

The second problem concerns the latest form of trauma exposure, the Chronic Indirect Vicarious Exposure, which is particularly frequent among first aiders, as it is considered in some studies as a risk factor for burnout (Boudoukha et al., 2013; Ordway et al., 2020). Burnout is a mental issue among workers characterised by three set of symptoms: exhaustion, cynicism and low efficacy (Maslach et al., 2001). Studies show that burnout, depression and PTSD are correlated and that burnout can either precede or succeed the exposure to or multiple TE among workers (Van Der Ploeg and Kleber, 2003; Mitani et al., 2006; Ehring et al., 2011). The prevalence of mild burnout in the general working population is 25% and severe burnout 2.7% (Honkonen et al., 2006). Consequently, the prevalence of all mental issues following a TE deserves further studies among first aiders. For example, Del Ben et al. (2006) consider that MDD is possibly higher in first responder cohorts.

Many studies have been devoted to the psychological outcomes among people who have been directly exposed to traumatic events; however, the mental health of caregivers, particularly first aiders, such as the volunteer rescuers, remains fairly neglected. In fact, very little research has been carried out on the mental and physical health of first aiders and post-traumatic outcomes after major traumas.

In contrast, studies of PTSD in professional healthcare workers have exploded, particularly in the face of pandemics, such as COVID-19, or Ebola and natural disasters, such as earthquakes.

A review looking at frontline medical staff in the face of COVID-19 (Benfante et al., 2020) found that their female population was more likely to be affected by post-traumatic stress symptoms. It would, therefore, be interesting to see whether this gender bias is present in our sample. Additionally, the study showed that those with less experience showed more symptoms of PTSD during the pandemic; thus, we will look at the role of experience in our study. Ambulance workers suffer from myriad mental health problems, including 11% with PTSD symptoms and 15% showing anxiety and depression (Petrie et al., 2018). Frontline workers in pandemics (Ebola, SARS, H1N1, COVID-19 etc.) similarly show symptoms of PTSD, burnout, anxiety and depression and stress (Busch et al., 2021). We can therefore postulate that first aiders will show similar levels of psychopathology.

Our current study is inspired by this body of work to look at volunteer first aiders. First aiders exposed to vicarious or secondary TE can develop a tendency to blame victims, creating a distance between themselves and those experiencing primary direct TE and become cynical towards their work. Palm et al. (2004) also suggested that first aid responders working with those directly affected by trauma can show changes in self-identity, worldview, spirituality and general psychological functioning. This is a direct contradiction with the role of a first responder (Palm et al., 2004).

In the course of their duties, all civilian volunteers may be directly or vicariously exposed to multiple traumatic events (Palm et al., 2004). In light of these sporadic but alarming facts, a better understanding of first aid volunteers’ mental health seems to be required. Our study looks at the *Protection Civile*, the leading French association of first responder volunteers (with the exception of firefighters). This service recruits 32,000 members in 98 departments, both in mainland France and overseas territories. The *Protection Civile* undertakes three main tasks: teaching first aid techniques to the general public, providing first aid at major sporting and cultural events and being available to provide frontline assistance during major disasters.

Through this study, we will look to quantify levels of mental ill health in the voluntary first aiders group who experience chronic vicarious traumatic events in the course of their volunteering. The link between the traumatic events exposure and psychological outcomes will be studied in order to provide precious information to improve early assessment and better inform those designing training and mentoring primary and secondary prevention programmes.

## Materials and Methods

### Participants

The questionnaire from which our results were extrapolated was distributed among five departmental branches of the French *Protection Civile* service. As we are relying on third party dissemination, it is impossible for us to know how many first aiders were approached and are therefore unable to calculate a response rate.

Fifty-three first aiders (21 females and 32 males; *Mean* age = 32.4 years, *SD* = 13.6 years) were enrolled in our study. Volunteers were presented with an ethical consent form before being provided with an anonymous code. Participants reported on average 11 years of work experience as first aiders (*SD* = 8.79 years). Of the sample, 67.9% were single, widowed or divorced, and 32.1% were married or coupled, 52.9% had a high school diploma or less, whereas 47.2% had completed tertiary education courses.

A total of 58.5% of the sample reported having been exposed to at least one traumatic event in the course of their volunteering duties. According to the frequency of trauma encounters reported, we create two groups: the *more exposed* participants (reporting 10 or more traumas corresponding to the third quartile of the distribution; 26.4% of the total sample) and the *less exposed* participants (reporting fewer than 10 events; corresponding to the first quartile of the distribution; 73.6% of the total sample).

### Measures

#### Traumatic Events—IET

The inventory of traumatic events (IET; Ouagazzal and Boudoukha, 2019) is an inventory that allows the identification of the occurrence of 26 different traumatic events within four categories—catastrophes (e.g. floods, fires or explosions), accidents (e.g. car accidents, other transport accidents or serious accidents at work), voluntary violence (e.g. sexual violence with penetration, armed aggressions or war and combat-based trauma) and death (e.g. homicide, the suicide of another individual or the brutal death of a close family member). By reference to each event, the respondent is asked to evaluate its current distress on an 11-point scale from 0 (no current distress) to 10 (extreme distress). The inventory permits to differentiate between trauma in private life and professional trauma.

#### PTSD—Checklist for DSM-5 (PCL-5)

The PCL-5 is a 20-item self-report measure that assesses the 20 DSM-5 symptoms of PTSD and therefore examines intrusions, avoidance, negative alterations in mood and cognitions and hyper-arousal (Weathers et al., 2013). By referencing each item, participants are required to rate their distress on 4-point Likert scales from 0 (not at all) to 4 (extremely). A cut-off score of 31 was used following the French validation of the PCL-5 (Ashbaugh et al., 2016). In the current sample, Cronbach’s *α* is 0.90.

#### Dissociative Experience Scale

The Dissociative Experience Scale (DES; Bernstein and Putnam, 1986) was adapted for digital use giving a scale for each question of 0–10. There are 28 questions, and the total DES scores were calculated by taking the average score and multiplying it by 10. Scores over 30 were considered as clinically significant in concordance with a French validation (Darves-Bornoz et al., 1999) and the *α* = 0.97.

#### Hospital Anxiety and Depression Scale

This scale originally developed by Zigmond and Snaith (1983) and provides two sub-scores—HADS-A, which measures anxiety and HADS-D, which measures depression. It comprises 14 questions (seven per sub-score) with multiple choice. Each question can be answered on a 4-point scale whose meaning can vary between questions e.g., “I feel happy”, possible answers between 3 (Not at all) and 0 (Most of the time). The French validation was demonstrated in a working population (Bocéréan and Dupret, 2014), and a score equal or superior to eight on each scale is seen as clinically significant and the *α* = 0.77 for hospital anxiety and depression scale (HADS)-A and *α* = 0.77 for HADS-D.

#### Alcohol Use Disorder Identification Test

The alcohol use disorder identification test (AUDIT) is a screening instrument for alcohol misuse developed by the WHO (Saunders et al., 1993). It comprises 10 multiple-choice questions with answers ranging from 0 (no consumption of alcohol) to 4 (signs of alcohol abuse). A threshold of eight was used following the original validation article as this provides the highest sensitivity. The French validation was used (Gache et al., 2005), that in this study shows a good internal consistency (*α* = 0.72).

#### Tobacco Use—Fagerström Test for Nicotine Dependence

This screening tool (Heatherton et al., 1991) comprises six questions with a total possible score of 10. The cut-off divides respondents into five groups—no dependence for those scoring lower than 2, low dependence for scores of 3 or 4, medium dependence for scores of 5 or 6, high dependence for 7–8 and very high dependence for those scoring 9 or 10. A French validation was used (Pomerleau et al., 1994) with an *α* = 0.74.

#### Burnout—Oldenberg Burnout Inventory

The Oldenberg Burnout Inventory (OLBI; Demerouti et al., 2003) measures exhaustion and disengagement on two subscales of eight items with a total burnout score available by adding the two. Each question has four possible answers ranging from 1 (totally disagree) to 4 (totally agree). Following the French validation (Angenot and Hansez, 2013), there were three groups—low (under 30/64), medium (between 30 and 45/64) and high burnout (45 or more/64). The internal consistency is *α* = 0.89.

#### Eating Disorders—The Sick, Control, One Stone, Fat, Food Questionnaire

The Sick, Control, One Stone, Fat, Food (SCOFF) questionnaire (Morgan et al., 1999) is comprised of five yes/no questions covering anorexia nervosa and bulimia nervosa symptomatology. A score of 2 or more is indicative of a clinically significant problem. A French validation was used (Garcia et al., 2010). The internal consistency was poor (*α* = 0.33).

#### Obsessive Compulsive Disorder—Maudsley Obsessive Compulsive Inventory (Abbreviated; MOCI-20)

This scale is a French validation (Hantouche et al., 2003) of a 20-item version of the MOCI-30. The inventory is comprised of 20 yes/no questions with a total possible score of 20. A score of 6 or higher is indicative of obsessive–compulsive symptomatology. Cronbach’s alpha was satisfactory at *α* = 0.80.

### Procedure

Email contact was sought with all *Protection Civile* departmental sections whose email address was available. Five sections responded and forwarded our invitation letter to their first aiders. Their participation was entirely voluntary, and no monetary reward was given. The questionnaires were all digitised and available on a Lime Survey site. The first aiders form a subpopulation of emergency workers, and it is therefore impossible to calculate the attrition rate. Ethical approval was given to this study by the University of Nantes (SY/GD/CB 2021 DRPI n°143).

Data were analysed with The jamovi project (2021). Due to the non-normality of the sample, we used a series of non-parametric tests, primarily Spearman’s rho for correlations as well as Mann–Whitney’s U for the sex variable and high/low reporters. In addition, we performed a series of *X*^2^ in order to compare a small selection of mental health variables (PTSD, burnout, alcohol and eating disorders) to the general population.

## Results

### Mental Health of First Aiders

Females showed higher scores of PTSD than males (respectively, *Mdn-female* = 19 *Mdn-male* = 7; *U =* 208.0, *p =* .02). No other gender difference in mental health outcomes, levels of education and marital status was found. Age was correlated with nicotine dependence (*r_s_* = 0.622, *p* = .023) with older participants reporting heavier use of cigarettes, and with obsessive–compulsive symptom (OCS) severity, (*r_s_* = −.330, *p* = .016) with younger participants reporting more severe OCS. As expected, age and experience positively correlated (*r* = .805, *p* < .001).

Correlations between mental health outcomes are reported in Table 1. PCL-5 scores were significantly correlated with all variables apart from the Fagerström Test for Nicotine Dependence (FTND) and the SCOFF. HADS-A statistically significant correlation with all the variables apart from FTND and HADS-D.

**Table 1:** Descriptive statistics, Cronbach’s *α* and Spearman’s rho correlations between mental health measures.

S. No.		N	Mean (SD)	*α*	Clinical prevalance	1	2	3	4	5	6	7	8
1.	HADS-A	53	6.51 (2.60)	.77	30.2%	–							
2.	HADS-D	53	4.42 (3.44)	.77	15.1%	.198	–						
3.	PCL-5	53	13.1 (11.2)	.90	28.3%	.540^***^	.362^**^	–					
4.	SCOFF	53	.91 (.99)	.33	28.3%	.354^**^	.309^*^	.155	–				
5.	MOCI	53	2.30 (2.79)	.80	11.3%	.954^***^	.216	.536^***^	.334^*^	–			
6.	AUDIT	30	5.03 (3.35)	.72	23.3%	.390^*^	.130	.374^*^	.133	.291	–		
7.	FTND	13	2.85 (2.48)	.74	53.9%	.095	−114	.261	.060	.049	.035	–	
8.	DES	53	14.4 (17.6)	.97	13.2%	.281^*^	.291^*^	.536^***^	.257	.451^***^	.229	.251	–
9.	OLBI	53	35.7 (8.97)	.89	Medium 64.6% High 13.2%	.533^***^	.590^***^	.330^*^	.413	.398^**^	.332	.320	.145

* *p* < .05;

** *p* < .01;

*** *p* < .001.

### Trauma Exposure and Mental Health

There was no correlation between the absolute frequency of traumatic events reported and any of the mental health measures. A series of individual samples tests (Mann–Whitney’s U) was performed, comparing the high event reporters and low event reporters with no significant differences between the two groups for any of the psychopathology outcomes. There was a significant correlation between the number of traumatic events reported and the years of experience, *r_s_* = .315, *p* = .022.

The intensity of the traumatic event per traumatic event was also not significantly correlated with any of the mental health outcome variables. Experience was correlated significantly with the measures of obsessive–compulsive disorder (MOCI), *r_s_* = −.281, *p* = .041 and nicotine dependence (FTND), *r_s_* = .710, *p* = .007.

There were moderately strong relationships between the PCL-5 and the HADS-A, *r_s_* = .540, *p* < .001, and the DES, *r_s_* = .536, *p* < .001. Similarly, the HADS-A shares a moderately strong correlation between the MOCI, *r_s_* = .594, *p* < .001, and the OLBI, *r_s_* = .553, *p* < .001. Finally, there is also a moderately strong relationship between the HADS-D and the OLBI, *r_s_* = .590, *p* < .001, and between the MOCI and the DES, *r_s_* = .451, *p* < .001. Descriptive statistics of mental health variable scores are given in Table 1.

Prevalence rates of mental health disorders screened for using the scales used in this study were calculated using the cut-offs specified in the methods section of this article and can be seen in Table 1 and can be found alongside their prevalence in the general population in Table 2.

**Table 2:** Prevalence of mental health outcomes in our study and in the general population.

Mental health outcome	Prevalence in our study	Prevalence in general population
PTSD	11.3%	2.32% (Ohayon and Shapiro, 2000)
Anxiety	30.2%	5.1% (Wittchen, 2002)
Depression	15.1%	4.9% (Blazer et al., 1994)
OCD	11.3%	3% (CilliÇilli et al., 2004)
Eating disorder	28.3%	9% (Smink et al., 2012)
Dissociation	13.2%	9.7% (Johnson et al., 2006)
Alcohol dependence	23.3%	4.65% (Grant et al., 2004)
Nicotine dependence	53.9%	9.7% (Breslau et al., 2001)
Burnout (severe)	13.2%	2.7% (Breslau et al., 2001)

## Discussion

Mental health among first aiders: a ‘not so strong’ population?

All mental health disorder prevalence levels gleaned from our study were higher than those expected in a community sample. The prevalence of PTSD (11.3% vs. 2.32% in the general population; Ohayon and Shapiro, 2000), *X*^2^ (1, *N* = 236) = 11.9, *p* < .001 is high enough to warrant further study. It is possible that the PTSD refers to traumas experienced in their private lives and full-time occupations, but if that were the case, one would expect the prevalence to be close to that of the general population. Therefore, it is probable that we either have an accumulation of micro-traumas as detailed before or a self-selected population with higher levels of psychopathology. This suggests that first-aid volunteers present an important level of mental health symptoms. Given the design of the current study, however, a general response bias cannot be excluded. Further studies with control measures that are not expected to be affected in our sample are therefore suited. It is also important to bear in mind the small sample size. It was a case of convenience sampling, and there were a high number of dropouts on the questionnaire due to its length, so it is equally probable that we have a small self-selected population. It is impossible to calculate an attrition rate as they are a subpopulation of a larger study, and we are unable to extrapolate the drop-out level.

The female population showed higher scores of PTSD than their male counterpart. This is consistent with previous research (Benfante et al., 2020).

There was a moderately high correlation between the risk of experiencing a predisposition to dissociative symptoms and PTSD, as can be expected, but it is interesting to note the link between dissociation and OCD. This has been previously documented and is found to be most strongly linked to checking compulsions and obsessive intrusions (Watson et al., 2004). Anxiety was correlated with almost all the variables except depression, which is surprising. The lack of correlation between depression and anxiety is possibly due to the fact that these two scores are measured by two orthogonal dimensions and are therefore not correlated. Anxiety was particularly strongly linked to OCD, which is concordant given that both conditions (GAD and OCD) were present with high levels of anxiety; OCD and GAD have been found to be strongly comorbid and are probably convergently correlated. Indeed, the moderately strong correlation between anxiety and burnout can also be explaining by an overlap in symptoms, as patients with burnout have a lower ability to cope with anxiety (Diestel and Schmidt, 2010). The large amount of correlations across mental health outcomes is indicative of trans-diagnostic processes, such as cognitive biases and avoidance (Fusar-Poli et al., 2019).

Addiction and OCD: coping and emotion regulation among first aiders.

Alcohol abuse is highly prevalent [23.3% vs. 4.65% in the general population, *X*^2^ (1, *N* = 236) = 4.1, *p* < .001] and moderately strongly correlated to both anxiety and PTSD. Therefore, the theory can be put forward that alcohol plays a role in self-medication (Robinson et al., 2009). The prevalence of eating disorders is three times that of the community sample [28.3% vs. 9%; *X*^2^ (1, *N* = 236) = 1,165 *p* < .001]. The SCOFF screens for anorexia nervosa and bulimia nervosa. The cut-off is very low (two or more items out of five) and while this has been found to be highly specificity and low sensitivity in a previous study (Solmi et al., 2015), causing a large number of false negatives, it is possible that our sample has low specificity (false positive) and high sensitivity (false negative). Therefore, a higher cut-off score would be advisable.

Age and experience are highly correlated, as can be expected, and it is therefore not surprising that they are both correlated only with obsessive–compulsive symptomatology and nicotine dependence.

In the case of nicotine dependence, as it is not correlated with any other psychopathological outcomes, one can postulate that it is a social activity rather than a coping mechanism. Therefore, it is possible that this increases with experience and familiarity. An increase in the prevalence of OCD with age has been previously documented (Cath et al., 2017). Experience was significantly correlated with the number of traumatic events reported, due probably to the accumulation of traumatic events over time.

Chronic Indirect Vicarious Exposure at work: to depressive and burnout outcomes?

We must also look further at the high levels of burnout [64.2% with a medium level of burnout and 13.2% a high level vs. 25 and 2.7% r *X*^2^ (2, *N* = 236) = 11.9, *p* < .001 in the general population]. Equally, the moderately strong link between depression and burnout is not surprising, as there is an overlap between those mental issues (Bianchi et al., 2015), given that the emotional exhaustion is common to both disorders. It has been suggested that depression is a consequence of untreated burnout (Boudoukha, 2020). This burnout reflects high levels of emotional exhaustion and disengagement and needs to be studied from a voluntary organisational viewpoint. Either there is not enough psychosocial support being provided, working conditions are unsatisfactory or both. It is surprising that burnout is not correlated with age or experience and shows that it is not just linked to an accumulation of traumatic events, in direct contradiction. When considering burnout, one must remember that these are volunteers. It has been reported that working as first aiders during an emergency, a serious disaster or a major crisis can be accompanied by a sense of empowerment and coping potential for the individual. This has been considered a coping strategy and a means of achieving personal growth in the face of adversity (McFarland and Alvaro, 2000). Jaffe et al. (2012) showed that occasional first aiders (such as volunteers) who were not motivated by belonging to a certain social group were more prone than other volunteers to develop symptoms of post-traumatic stress disorder (PTSD). This result is consistent with the idea that social support is a protective factor in the face of adversity. In contrast, volunteering motivated by a desire for professional advancement or to improve one’s self-esteem is associated with a greater occurrence of psychopathological symptoms (El-Gabalawy et al., 2020).

### Limitations

It is surprising that not one mental health outcome correlated with the total number of traumatic events reported. It is possible that this is due to the small number of participants reporting a traumatic event as well as the extreme range of responses (0–294). As the prevalence of PTSD is so high, it is likely that some sort of trauma is being experienced. It is probable that there is a phenomenon of both under and over-reporting. It is also possible that the volunteers have an accumulation of ‘micro-traumas’ (Seides, 2010)—rather than large scale traumatic events—which would not be picked up by our inventory.

It is essential to mention the current pandemic and how it affects the results. Although first aiders were not on the frontline, they still volunteered in vaccination centres, working on logistics. It would have been interesting to have a pre- and post-pandemic measures.

## Conclusion

While the current study indicates that mental illness is prevalent among first aiders, a longitudinal study is necessary in order to draw more firm conclusions on whether trauma has a latency period before causing symptoms and thus be correlated with mental health outcomes. Looking at the high level of PTSD and burnout (as well as anxiety and depression, which are highly prevalent in this population) highlights the need for more psychosocial support with the potential to introduce the role of a liaison psychologist trained in trauma and first responder populations. There is also a role to play in peer support and creating cohesive teams through training, especially in mental health first aid, already taught in relation to possible patients but which could also be introduced to peer support, such as in the case of Pekevski (2013) who introduced psychological first aid to frontline staff.

While bearing in mind the limitations of the current study, it shows that volunteer first aiders are a neglected group with significant levels of psychopathology. There is a need for improved training and sustained support of this vulnerable group. It would also be interesting to introduce a module on resilience training to increase a shared organisational identity and develop and maintain leadership roles.

## Data Availability Statement

The raw data supporting the conclusions of this article will be made available by the authors, without undue reservation.

## Ethics Statement

The studies involving human participants were reviewed and approved by the CERNI (Ethical and Non Interventional Research Committee of the University of Nantes) DRPI N°143. The patients/participants provided their written informed consent to participate in this study.

## Author Contributions

CR: data analysis and writing of draft manuscript. GC: proofreading of manuscript and statistical advice. AB: ethical approval and supervision. All authors contributed to the article and approved the submitted version.

## Conflict of Interest

The authors declare that the research was conducted in the absence of any commercial or financial relationships that could be construed as a potential conflict of interest.

## Publisher’s Note

All claims expressed in this article are solely those of the authors and do not necessarily represent those of their affiliated organizations, or those of the publisher, the editors and the reviewers. Any product that may be evaluated in this article, or claim that may be made by its manufacturer, is not guaranteed or endorsed by the publisher.
